# Seasonal variation in the incidence of vitreous hemorrhage in proliferative diabetic retinopathy

**DOI:** 10.1371/journal.pone.0355497

**Published:** 2026-08-07

**Authors:** Mirinae Kim, Young-Hoon Park

**Affiliations:** 1 Department of Ophthalmology and Visual Science, College of Medicine, The Catholic University of Korea, Seoul, South Korea; 2 Seoul St. Mary’s Hospital, Seoul, South Korea; Akita University: Akita Daigaku, JAPAN

## Abstract

**Purpose:**

To investigate whether seasonal alterations and meteorological factors are associated with the incidence of vitreous hemorrhage in patients with proliferative diabetic retinopathy (PDR).

**Materials and Methods:**

This retrospective observational study included patients with PDR who visited Seoul St. Mary’s Hospital between January 2019 and December 2021. Among the 4,402 eyes with PDR, the presence of vitreous hemorrhage was determined. The monthly incidence of vitreous hemorrhage and meteorological data were obtained and analyzed.

**Results:**

Among 4,402 eyes of PDR, vitreous hemorrhage was observed in 293 eyes (6.66%). The incidence of vitreous hemorrhage was high in May (9.37%, 31 of 331 eyes) and June (8.58%, 26 of 303 eyes). Poisson regression analyses revealed that temperature, humidity, and precipitation-per-hour did not influence the risk of vitreous hemorrhage. Thus, the observed seasonal variation in vitreous hemorrhage was not fully explained by the measured meteorological factors. The Wilcoxon signed-rank test revealed a significant difference between the intraocular pressure (IOP) at the time of hemorrhage (hemorrhage-IOP; hot season) and IOP at the visit immediately before the occurrence of hemorrhage (pre-IOP; cold season) groups (P = .029) and between the hemorrhage-IOP (warm season) and pre-IOP (cold season) groups (P = .041).

**Conclusion:**

Vitreous hemorrhage in PDR showed seasonal variations, peaking in May and June. This pattern could not be fully explained by the meteorological variables evaluated; IOP-fluctuations caused by temperature changes and systemic factors such as seasonal changes in glycemic control may contribute to this variability. Further studies are warranted to elucidate the underlying mechanisms.

## Introduction

Proliferative diabetic retinopathy (PDR) is a relatively common cause of vision loss in patients with diabetes mellitus that affects approximately 7% of these patients. Diabetic retinopathy (DR) progresses from nonproliferative to proliferative stages, during which fragile neovascularization on the surface of the retina develops. These abnormal vessels are fragile and prone to rupture, leakage, and traction. The reported risk factors for vitreous hemorrhage in patients with PDR include poor glycemic control, severity of DR, extent of neovascularization, cerebrovascular disease, anticoagulant use, and a history of previous vitreous hemorrhage. [1–3]

Seasonal variations are observed in several ocular diseases. For instance, the incidence of retinal vein occlusion [4,5] and disc hemorrhage [6] is higher in winter. In contrast, rhegmatogenous retinal detachment shows a summer peak and no seasonality. [7–12] The incidence of recurrent wet age-related macular degeneration (AMD) is higher during the warm season owing to the higher temperatures and increased ultraviolet radiation. [13] However, acute submacular hemorrhage associated with AMD shows a winter-dominant incidence [14] or no seasonality. [15,16] Similarly, central serous chorioretinopathy has a higher incidence in spring. [17]

To the best of our knowledge, a consensus on seasonal variations in the incidence of vitreous hemorrhage in patients with PDR is lacking. Given that vascular factors such as retinal ischemia and neovascularization are involved in the pathogenesis of PDR, PDR may show seasonal variations. However, reports on seasonal variation in or association of PDR severity with meteorological factors are lacking. Identifying the factors influencing seasonal variations in hemorrhagic events in PDR would lead to better understanding of the pathophysiology of PDR. This study aimed to investigate the association of seasonal alterations and meteorological factors with the onset of vitreous hemorrhage in patients with PDR.

## Materials and Methods

This study was conducted in accordance with the principles of the Declaration of Helsinki and approved by the Institutional Review Board at Catholic University of Korea (approval number: KC24RISI0617). Data were accessed for this study from September 20th, 2024 to May 20th, 2025 in accordance with IRB approval. The requirement for written informed consent was waived because of the retrospective nature of this study.

This retrospective cohort study included all patients with PDR who were followed up at Seoul St. Mary’s Hospital and underwent fundus photography between January 2019 and December 2021. Based on the fundus photographs, the presence of vitreous hemorrhage was determined by a masked observer (H.S.). Vitreous hemorrhages that persisted since the previous visit were not recorded as new hemorrhages. Patients with intraocular hemorrhage unrelated to DR were excluded.

Demographic information and medical histories were collected from the medical records. For patients with hemorrhage on fundus photographs, blood test results within 1 month from the date of hemorrhage and systolic/diastolic blood pressure on the day of hemorrhage were retrieved from the medical records. To determine the effect of intraocular pressure (IOP) or IOP fluctuations on the incidence of hemorrhage, the IOP on the day of hemorrhage (H-IOP) and the IOP at the visit immediately before the occurrence of hemorrhage (pre-IOP) were retrieved from the medical records.

Climatological information was downloaded from the Korea Meteorological Administration website (www.weather.go.kr). We collected information on regional and monthly average temperatures, precipitation, and humidity according to the patients’ residential areas. We split each calendar year into three seasons according to the average monthly temperature: T ≥ 20°C (Hot), 10°C ≤ T < 20°C (Warm), and T < 10°C (Cold). The H-IOP and pre-IOP groups were subdivided into three groups according to the temperature subgroups (Hot, Warm, and Cold groups).

All statistical analyses were conducted using SPSS software (version 29.0; IBM Corp., Armonk, New York, USA). *P* value under 0.05 was considered to indicate statistical significance. After confirming the normal distributions of the variables, the relationship between pre-IOP and H-IOP was analyzed using the Wilcoxon signed-rank test. The mean temperature, humidity and precipitation per hour, and month were used to predict the occurrence of vitreous hemorrhage by Poisson regression.

## Results

Among 4,402 eyes that underwent ophthalmic examinations between January 2019 and December 2021, vitreous hemorrhage was confirmed in 293 eyes (6.66%). The mean age of patients was 56.7 ± 11.8 years, and 139 (59.9%) were male. Seventy-two patients (31.0%) had hypertension. Among 293 eyes with vitreous hemorrhage, 9 (3.9%) were followed up without any treatment, 143 (61.6%) received laser photocoagulation, 192 (82.8%) received intravitreal injection of anti-vascular endothelial growth factor, and 45 (19.4%) required surgical treatment (Table 1).

**Table 1:** Baseline demographics and clinical characteristics of study participants.

Patient characteristics	Value
Age, years	56.7 ± 11.8
Sex, n (%)	
Female	93 (40.1%)
Male	139 (59.9%)
Laterality, n (%)	
Right eye	112 (48.3%)
Left eye	120 (51.7%)
DM years	12.3 ± 3.3
Underlying disease	
Hypertension	72 (31.0%)
Chronic kidney disease	33 (14.2%)
Cardiovascular disease	44 (19.0%)
Best-corrected visual acuity (logMAR)	0.97 ± 0.75
Intraocular pressure (mmHg)	16.3 ± 3.3
HbA1c	7.48 ± 1.23
Fasting glucose	133.26 ± 42.67
Serum creatinine	1.60 ± 2.73
eGFR	73.59 ± 31.13
Systolic BP	129.94 ± 17.40
Diastolic BP	75.08 ± 9.22
Treatment	
None	9 (3.9%)
Laser photocoagulation	143 (61.6%)
Anti-VEGF injection	192 (82.8%)
Surgical intervention	45 (19.4%)

DM, diabetes mellitus; logMAR, logarithm of the minimum angle of resolution; HbA1c, glycosylated hemoglobin; eGFR, estimated glomerular filtration rate; BP, blood pressure; VEGF, vascular endothelial growth factor.

**Fig 1:**
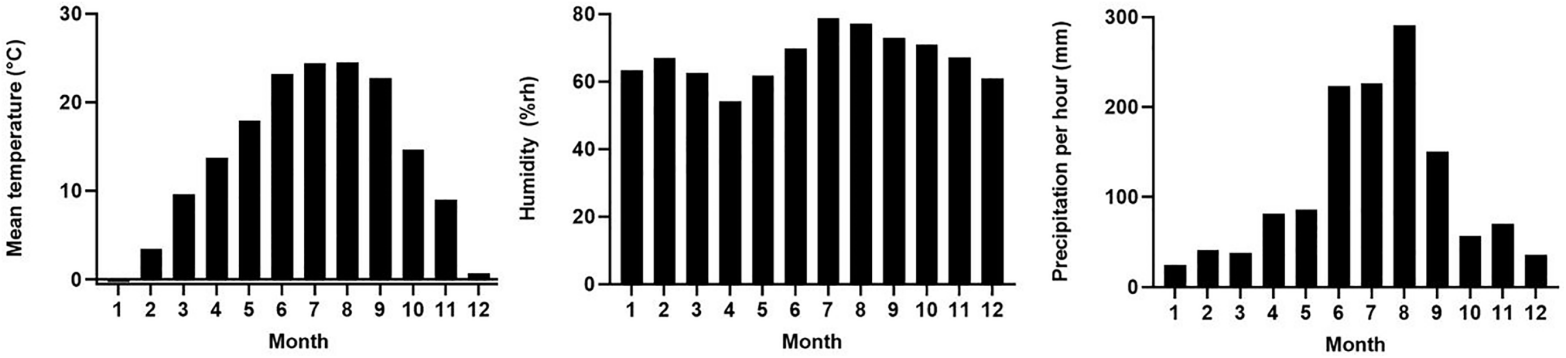
Monthly meterological information including mean temperature, humidity and precipitation per hour.

**Fig 2:**
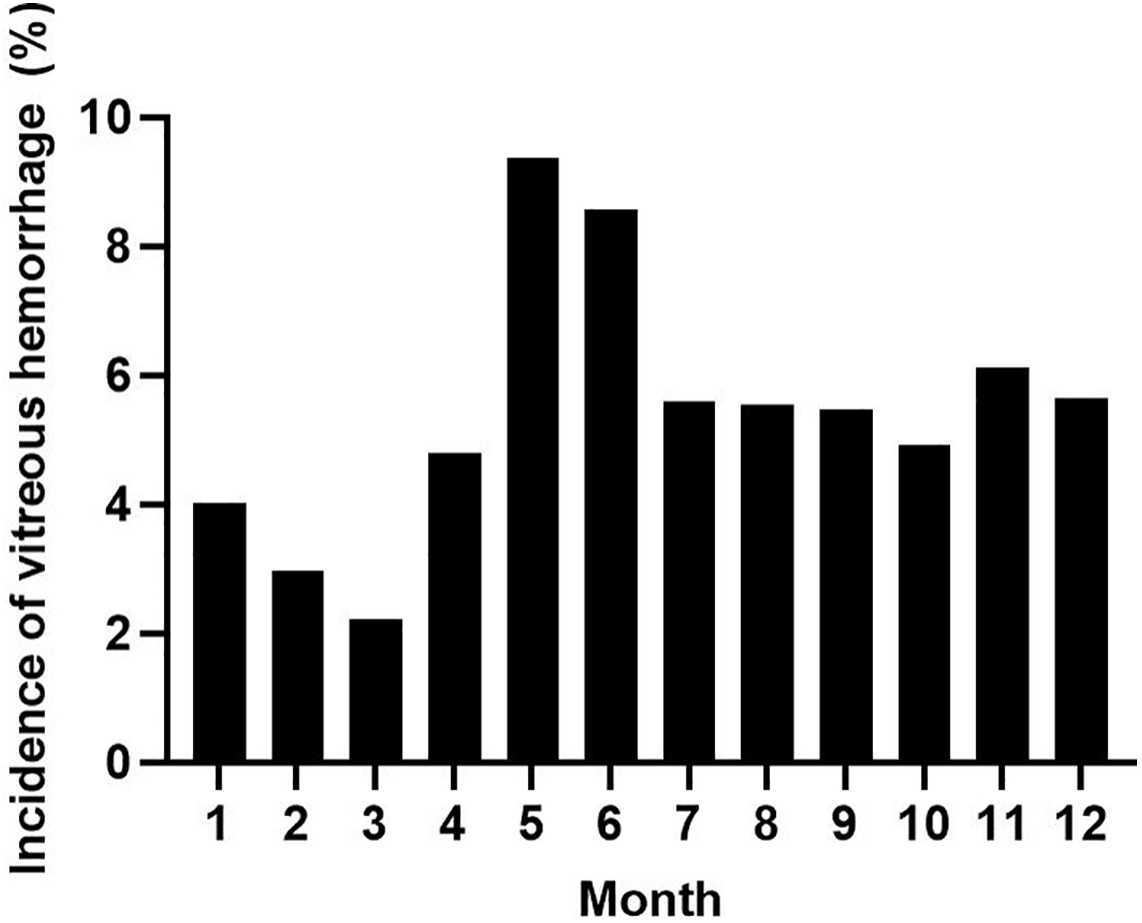
Monthly incidence of vitreous hemorrhage.

The monthly incidence of vitreous hemorrhage and meteorological information are shown in Fig 1, Fig 2 and Table 2. The percentage of vitreous hemorrhage was relatively high in May (9.37%, 31 of 331 eyes) and June (8.58%, 26 of 303 eyes). Poisson regression analyses revealed that for every 1°C rise of temperature was associated with a risk ratio of 1.348 (95% confidence interval [CI], 0.703–2.538); but not statistically significant (P= = .368; Table 3). Furthermore, for every 1% increase in humidity increased and 1 mm increase in precipitation per hour, the vitreous hemorrhage risk ratios increased by 1.104 (95% CI, 0.588–2.071; P= = .759) and 1.357 (95% CI, 0.703–2.619; P= = .363), respectively. The vitreous hemorrhage incidence rates in May and June were 2.104 (95% CI, 1.151–3.846, P= = .016) and 1.764 (95% CI, 0.946–3.289, P= = .044), respectively. Although our analyses did not show a statistically significant association between vitreous hemorrhage incidence and other climatological factors such as humidity and precipitation, the limited sample size and the possibility of confounding, including variation in the time interval between visits, preclude definitive conclusions.

**Table 2:** Monthly incidence of vitreous hemorrhage and meteorological information.

Month	Incidence of vitreous hemorrhage	Mean temperature (°C)	Humidity (%rh)	Precipitation per hour (mm)
January	4.02% (16/398)	−0.27 ± 1.71	63.43 ± 13.02	24.77 ± 12.58
February	2.98% (12/402)	3.47 ± 2.16	67.00 ± 7.21	41.27 ± 40.54
March	2.22% (10/451)	9.66 ± 2.33	62.60 ± 9.52	38.44 ± 42.29
April	4.80% (18/375)	13.77 ± 2.94	54.22 ± 3.17	81.79 ± 50.69
May	9.37% (31/331)	17.97 ± 5.19	61.83 ± 5.97	86.34 ± 66.25
June	8.58% (26/303)	23.22 ± 0.64	69.70 ± 4.73	223.53 ± 115.58
July	5.59% (19/340)	24.39 ± 4.81	78.75 ± 2.18	226.71 ± 83.18
August	5.54% (20/361)	24.49 ± 4.35	77.11 ± 5.65	290.53 ± 156.48
September	5.47% (21/384)	22.76 ± 0.46	72.89 ± 2.97	150.49 ± 26.99
October	4.92% (16/325)	14.63 ± 1.42	71.00 ± 7.51	57.15 ± 32.65
November	6.11% (22/360)	8.99 ± 3.78	67.13 ± 5.45	70.31 ± 6.22
December	5.65% (21/372)	0.72 ± 2.66	60.89 ± 6.33	36.03 ± 40.94

**Table 3:** Poisson regression analyses of hemorrhage incidence according to temperature.

Variable	Exp(b)	*P*–value
Temperature, per 1°C rise	1.348 (0.703–2.583)	.368
Humidity (%rh)	1.104 (0.588–2.071)	.759
Precipitation per hour (mm)	1.357 (0.703–2.619)	.363
		
Month		
January	Reference	
February	0.713 (0.337–1.506)	.713
March	0.528 (0.240–1.163)	.528
April	1.221 (0.623–2.395)	.560
May	2.104 (1.151–3.846)	.016
June	1.764 (0.946–3.289)	.044
July	1.289 (0.663–2.507)	.454
August	1.357 (0.703–2.619)	.363
September	1.425 (0.744–2.731)	.286
October	1.086 (0.543–2.171)	.816
November	1.493 (0.784–2.842)	.223
December	1.348 (0.703–2.583)	.368

The relationship between IOP fluctuations and the incidence of vitreous hemorrhage is summarized in Table 4. The Wilcoxon signed-rank test indicated a significant difference between the H-IOP (Hot) and pre-IOP (Cold) groups (P = .029) and between the H-IOP (Warm) and pre-IOP (Cold) groups (P = .041). The frequency of vitreous hemorrhage was significantly higher in seasons where temperatures increased and in situations where IOP decreased.

**Table 4:** Analysis of intraocular pressure changes in each paired group.

Group		N	Mean	Paired difference	P value
1	H-IOP(Hot)	42	16.43 ± 3.16	−0.21 ± 2.93	.536
	Pre-IOP(Hot)		16.64 ± 2.65		
2	H-IOP(Hot)	22	16.27 ± 2.99	−1.04 ± 4.76	.280
	Pre-IOP(Warm)		17.20 ± 3.11		
3	H-IOP(Hot)	23	15.39 ± 3.96	−1.30 ± 3.17	.029
	Pre-IOP(Cold)		16.69 ± 2.24		
4	H-IOP(Warm)	18	17.33 ± 5.22	0.44 ± 3.32	.664
	Pre-IOP(Hot)		16.89 ± 3.70		
5	H-IOP(Warm)	20	15.79 ± 4.42	−0.78 ± 3.95	.659
	Pre-IOP(Warm)		16.57 ± 3.84		
6	H-IOP(Warm)	37	15.26 ± 3.10	−0.84 ± 2.69	.041
	Pre-IOP(Cold)		16.08 ± 2.05		
7	H-IOP(Cold)	21	15.24 ± 3.20	0.52 ± 2.80	.622
	Pre-IOP(Hot)		14.71 ± 2.53		
8	H-IOP(Cold)	16	16.56 ± 1.50	1.18 ± 3.19	.234
	Pre-IOP(Warm)		15.37 ± 2.19		
9	H-IOP(Cold)	33	15.97 ± 3.07	−0.61 ± 3.93	.210
	Pre-IOP(Cold)		16.57 ± 4.61		

IOP, intraocular pressure; H-IOP, IOP on the day of the hemorrhage; Pre-IOP, IOP at the visit immediately before the occurrence of hemorrhage; Hot, monthly average temperature ≥20°C; Warm, monthly average temperature ≥10°C but < 20°C; Cold, monthly average temperature < 10°C.

## Discussion

This study investigated the influence of seasonal and climatic changes on the incidence of vitreous hemorrhage in PDR patients. In this study, vitreous hemorrhage in PDR appeared to occur most frequently in May and June. However, this variation could not be fully explained by the measured meteorological factors including temperature, humidity, and precipitation.

Seasonal changes and weather have long been known as important triggering factors for various cardiovascular diseases. [18,19] Similarly, epidemiologic studies have reported that the incidence of retinal vascular disorders such as retinal artery occlusion, retinal vein occlusion, optic disc hemorrhage, and submacular hemorrhage, is higher in winter than in summer. [4,6,13,16,20,21] In contrast, the incidence of bacterial endophthalmitis after cataract and glaucoma filtering surgery increases during the warmer months [22,23] or shows no seasonal variation. [24] Cold temperature induces systemic vasoconstriction and increases the blood pressure. [25,26] The consequent increased oxygen demand, vascular dysregulation, and vasospasm of the retinal microvasculature could trigger retinal vascular diseases. DR is classified as a retinal vascular disease; however, vitreous hemorrhage in patients with PDR does not occur more frequently in winter. This suggests that vitreous hemorrhage is not caused by vasospasm alone. The risk factors for vitreous hemorrhage include poor glycemic control, severity of DR, extent of neovascularization, cardiovascular disease, and history of previous vitreous hemorrhage. Our findings suggest a possible association between IOP changes when moving from a low‑ to a high‑temperature environment and the risk of vitreous hemorrhage in patients with PDR; however, this interpretation is exploratory and may be influenced by the time‑dependent progression of PDR and other sources of confounding.

Furthermore, PDR is a progressive disease, and the risk of vitreous hemorrhage is likely influenced by the natural course of disease progression over time. In our analysis of nine temperature‑based groups (combinations of hot, warm, and cold conditions), we did not adjust for the variable time interval between the previous visit and the visit at which vitreous hemorrhage occurred. Therefore, the differences observed between temperature‑based groups may partly reflect differences in follow‑up duration rather than a true effect of temperature or IOP changes alone. In addition, our analyses did not demonstrate a clear or statistically significant association between incidence of vitreous hemorrhage and other climatological variables such as humidity and precipitation; however, these findings should be interpreted cautiously, as the current analysis does not provide sufficient evidence to support a causal or robust associative relationship.

One additional hypothesis regarding the higher frequency of bleeding in May and June is that the frequency of posterior vitreous detachment (PVD) may be higher during the warmer months. Seasonal variations in PVD are not well understood. However, some studies have suggested that vitreous shrinkage secondary to excessive sweating and dehydration at high temperatures also contributes to PVD. [8,27] In addition, the degree of glycemic control is affected by seasonal changes [28]; however, since this study did not continuously analyze serum glucose levels by season, additional research is needed.

Our study had several limitations. Because this study was retrospective in nature, uncontrolled confounding factors or selection bias may have significantly affected the results. In particular, because the follow-up interval differed among patients, the timing of the hemorrhage may not have been accurately recorded. The lack of adjustment for follow‑up interval, especially in the analysis using nine temperature‑based groups, may have caused differences between groups that reflect variation in follow‑up duration rather than the effect of temperature or IOP‑related changes alone. Additionally, we were unable to analyze other clinically relevant factors that may have influenced the incidence of vitreous hemorrhage. HbA1c is a well-established major risk factor for the progression of diabetic retinopathy; however, one of the main limitations of this study is that we were unable to repeatedly analyze seasonal changes in HbA1c or fasting glucose levels. In this study, vitreous hemorrhage was classified using conventional fundus photography, which was available for all patients, whereas widefield fundus photography was not available in a small subset of cases. Consequently, subtle or peripheral vitreous hemorrhage may have been missed, which could introduce selection bias. Despite the limitations of this study, the strength of our study is that it is the first to report seasonal variation in vitreous hemorrhage in patients with PDR. Recently, patient education has become important because of the development of self-monitoring tools such as wearable devices. Awareness regarding the circumstances that increase the likelihood of vitreous hemorrhage recurrence can be helpful in patient education.

In the management of PDR, knowledge about the seasonal variations in the incidence of vitreous hemorrhage may be important for preventing significant vision loss in patients with PDR. Our study demonstrated a peak incidence of vitreous hemorrhage in May and June. This variation could not be fully explained by the measured meteorological factors (temperature, humidity, and precipitation) and should be interpreted with caution. Further prospective studies with larger populations and more detailed environmental and clinical information are required to validate these observations and to elucidate the mechanisms underlying possible seasonal variation in vitreous hemorrhage among patients with PDR.
